# Supplementary figures and images for: The blood–brain barrier regulates brain tumor growth through the SLC36 amino acid transporter Pathetic in Drosophila
Source: PLoS Biol. 2025 Nov 18;23(11):e3003496. doi: 10.1371/journal.pbio.3003496 (PMC12626262; doi:10.1371/journal.pbio.3003496)

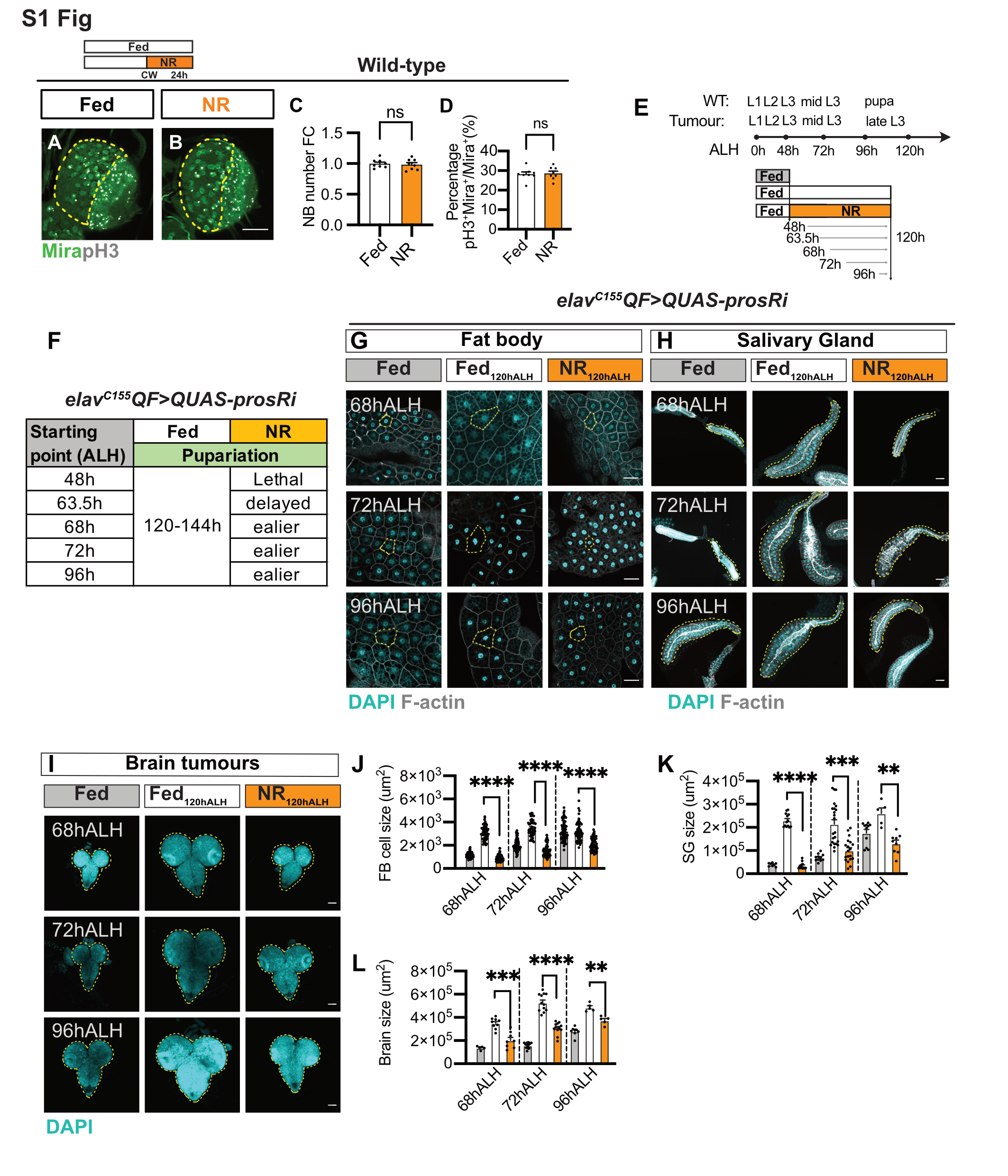

Supplement: S1 Fig — (A, B) Single-section images of wild-type brain lobes (ventral side) stained with the Miranda (Mira) and the mitotic marker pH3 under Fed and NR conditions. CBs are circled by yellow dashed lines. NR: 63.5-96hALH; Dissection: 96hALH. Genotype: repo-G4>UAS-RFP. (C, D) Quantification of type I NB number (based on Mira staining) and the percentage of type I NBs undergoing mitosis (pH3+Mira+) in the CB in (A, B) (n = 8, 8). (E) Schematic representation of the Fed/NR regime. (F) Table depicting the consequence of NR starting from 48, 63.5, 68, 72, and 96hALH on pupariation of elavC155QF>QprosRNAi tumor-bearing animals. (G–I) NR from 68, 72, and 96hALH significantly reduced the size of fat body cells, salivary glands, and brain tumors (circled by yellow dashed lines). (G, H): single-section images of fat body and maximum projection images of salivary glands, marked by DAPI and Phalloidin at NR starting time point, Fed120hALH and NR120hALH. (I): maximum projection images of brain tumors, marked by DAPI at NR starting time point, Fed120hALH and NR120hALH. Scale bar = 100 μm for (G) and (H). (J–L) Quantifications of the area of a single fat body cell circled in (G), the salivary gland circled in (H) and the whole brain circled in (I). In (J): n68hALH = 105, 60, 75; n72hALH = 79, 52, 68; n96hALH = 74, 62, 58. In (K): n68hALH = 8, 13, 12; n72hALH = 11, 23, 19; n96hALH = 11, 5, 9. In (L): n68hALH = 5, 9, 8; n72hALH = 13, 12, 12; n96hALH = 8, 5, 5. Genotype: elavC155QF>QprosRNAi. Data information: ALH = after larvae hatching. Scale bar = 50 μm unless otherwise stated. The error bar represents SEM. In (C): unpaired t test, (ns) P = 0.8060. In (D): unpaired t test, (ns) P = 0.7211. In (J): Kruskal–Wallis H Test, (****) P < 0.0001; Kruskal–Wallis H Test, (****) P < 0.0001; Kruskal–Wallis H Test, (****) P < 0.0001. In (K): Kruskal–Wallis H Test, (****) P < 0.0001; Kruskal–Wallis H Test, (***) P = 0.0002; Kruskal–Wallis H Test, (**) P = 0.0011. In (L): (***) P = 0.0006; Welch’s ANOVA [file pbio.3003496.s001.tiff]

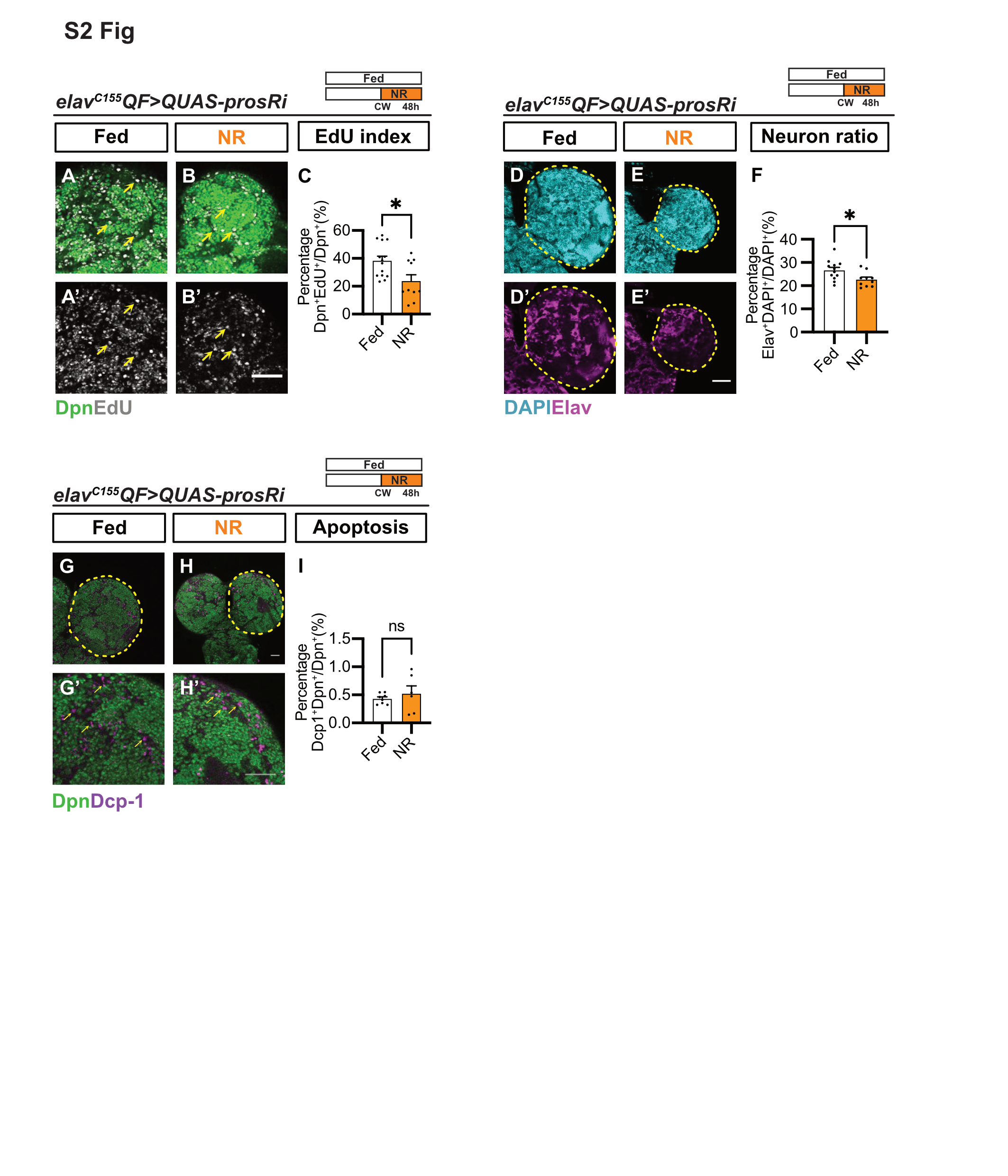

Supplement: S2 Fig — (A–B′) Single-section images of elavC155QF>QUAS-prosRi tumor brains labeled with EdU (yellow arrows) and Dpn under Fed and NR conditions (the same dataset from Fig 1J–1L). A and B: Dpn and EdU; A′ and B′: EdU. (C) Quantification of the percentage of NB tumors (Dpn+, green) that are EdU+ in each brain lobe of (A, B) (n = 14, 10). (D–E′) Single section images of elavC155QF>QUAS-prosRi tumor brains stained with DAPI and the neuronal marker Elav under Fed and NR conditions. (F) Quantification of the percentage of neurons among all brain cells in each brain lobe of (D–E′) (n = 12, 10). (G–H′) Single section images of elavC155QF>QUAS-prosRi tumor brains stained with Dpn and the cell death marker Dcp-1 (yellow arrows) under Fed and NR conditions. (G′) and (H′) are zoomed-in images of (G) and (H). (I) Quantification of the percentage of NBs (Dpn+) undergoing apoptosis (Dcp-1+) of each circled brain lobe in (G, H) (n = 8, 6). Data information: ALH = after larvae hatching. NR: 72-120hALH; Dissection: 120hALH unless otherwise stated. Brain lobes are circled with yellow dashed lines. Scale bar = 50 μm. Error bar represents SEM. In (C): unpaired t test, (*) P = 0.0128. In (F): unpaired t test, (*) P = 0.0247. In (I): Welch’s t test, (ns) P = 0.5374. Raw data are included in S3 Data. (TIFF) [file pbio.3003496.s002.tiff]

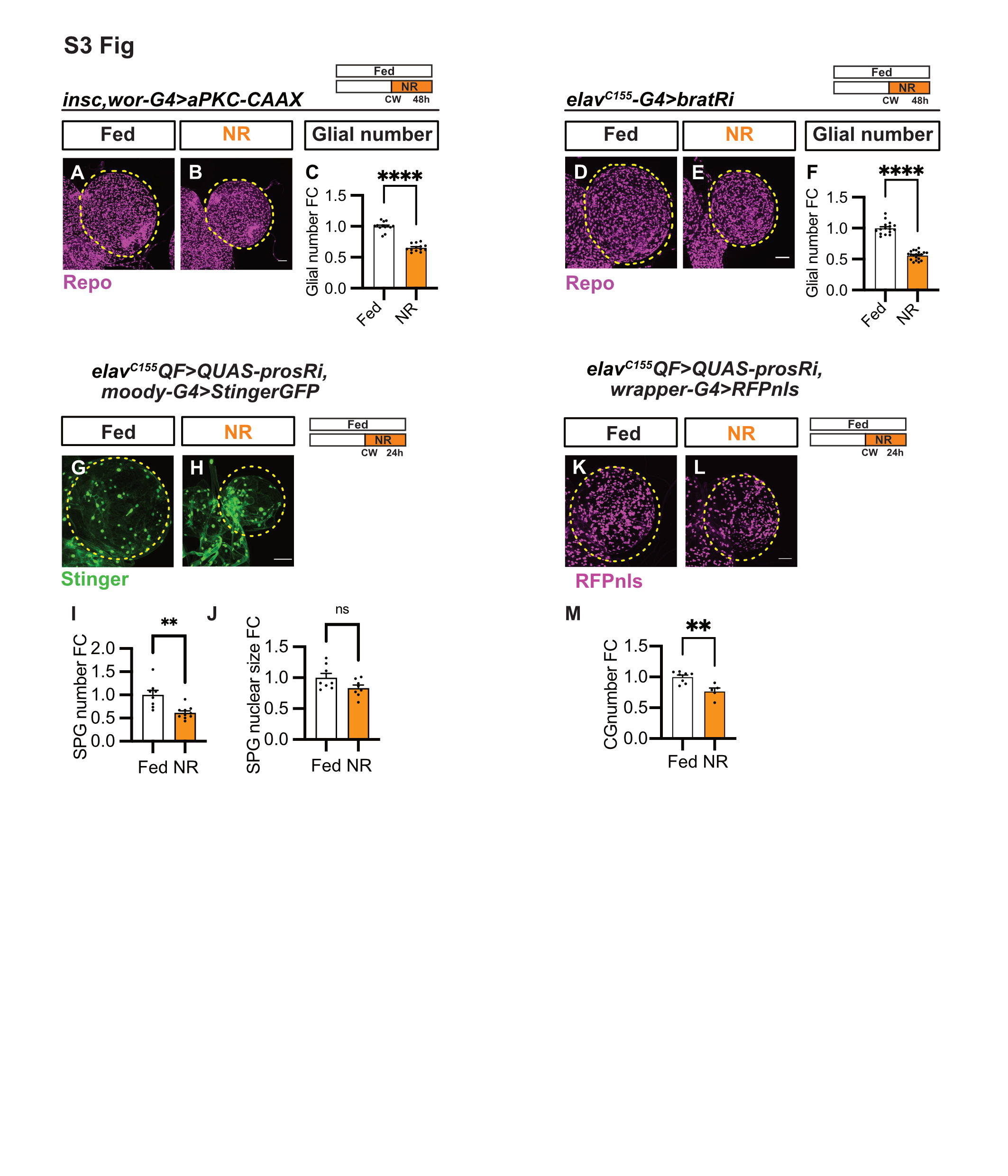

Supplement: S3 Fig — (A, B) Maximum projection images of insc-G4,wor-G4>aPKC-CAAX tumor brains stained with Repo under Fed and NR conditions. (C) Quantification of normalized (to Fed) glial number of circled brain lobes in (A, B) (n = 13, 13). (D, E) Maximum projection images of elavC155-G4>bratRi tumor brains stained with Repo under Fed and NR conditions. (F) Quantification of normalized (to Fed) glial number of circled brain lobes in (D, E) (n = 16, 20). (G, H) Single-section images of elavC155QF>QUAS-prosRi tumor brain lobes, where SPG are marked with moody-G4>UAS-Stinger, under fed and NR conditions. NR:72-96hALH. Dissection: 96hALH. (I) Quantifications of SPG numbers (normalized to fed) in (G, H) (n = 8, 10). (J) Quantification of SPG nuclear size (normalized to fed) in (G, H) (n = 8, 8). (K, L) Maximum projection images of elavC155QF>QUAS-prosRi tumor brain lobes, where CG are marked with wrapper-G4>UAS-RFPnls, under fed and NR conditions. NR:72-96hALH. Dissection: 96hALH. (M) Quantification of CG number (normalized to fed) in (K, L) (n = 8, 5). Data information: ALH = after larvae hatching. NR: 72-120hALH; Dissection: 120hALH unless otherwise stated. Brain lobes are circled with yellow dashed lines. Scale bar = 50 μm. Scale bar = 20 μm in K′, L′, and M′ Error bar represents SEM. In (C): unpaired t test, (****) P < 0.0001. In (F): unpaired t test, (****) P < 0.0001 In (I): Welch’s t test, (**) P = 0.0054. In (J): Welch’s t test, (ns) P = 0.069. In (M): unpaired t test, (**) P = 0.0021. Raw data are included in S3 Data. (TIFF) [file pbio.3003496.s003.tiff]

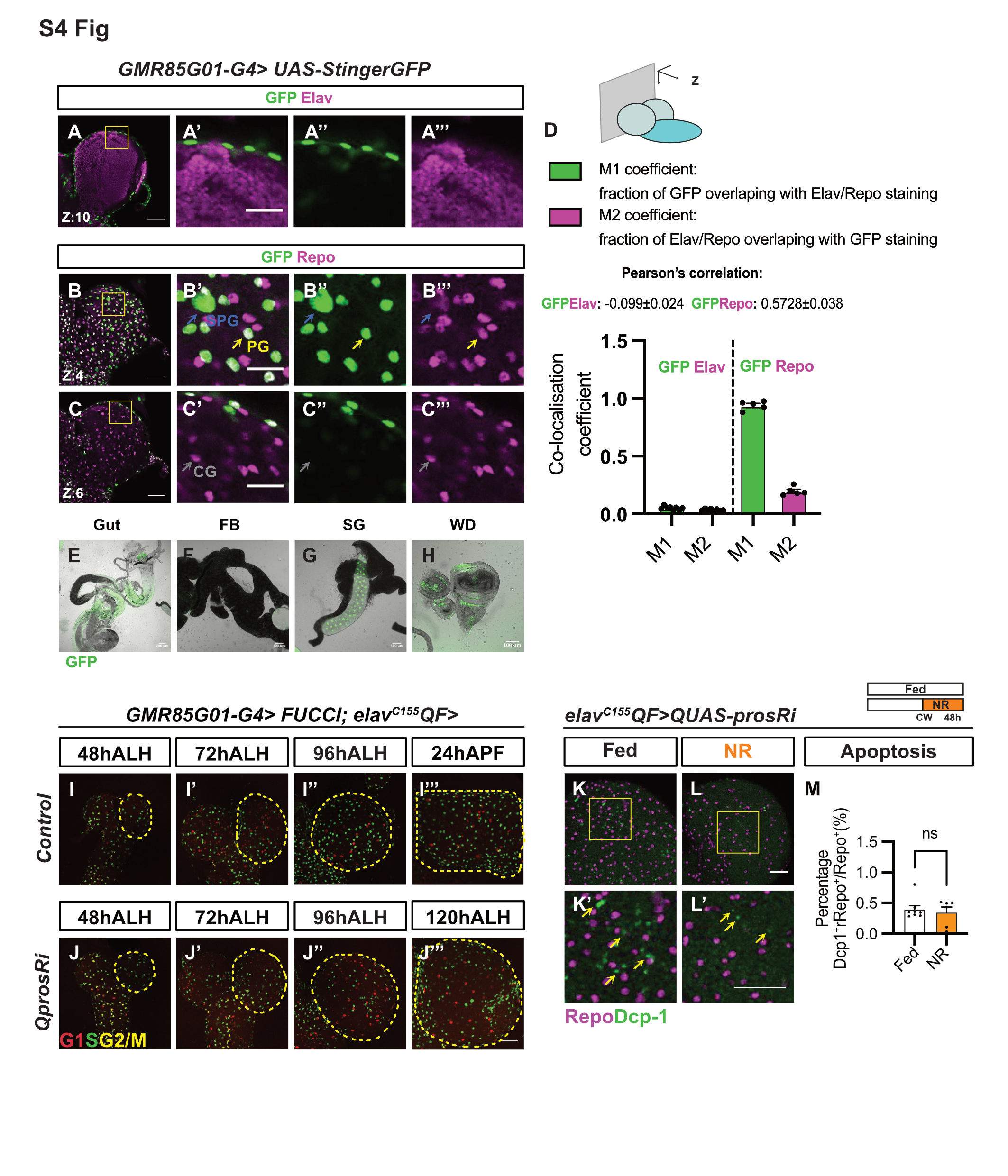

Supplement: S4 Fig — (A–A′″): Deep-section image of late L3 wildtype larval brain lobe, where GMR85G01-G4>UAS-Stinger showed no colocalization with neurons (marked by Elav in magenta). A′–A′″ are zoomed-in images of A. (B–B′″): Surface-section image of late L3 wildtype larval brain lobe, where GMR85G01-G4>UAS-Stinger colocalised with some glial cells (marked by Repo). These GFP+ glial cells include PG (yellow arrows) and SPG (with enlarged nuclei, caused by endoreplication, blue arrows). And those GFP- glial cells are surface-associated CG. B′–B′″ are zoomed-in images of B. (C–C′″): Mid-section image of late L3 wildtype larval brain lobe, where GMR85G01-G4>UAS-Stinger did not colocalise with the glial cell marker Repo. These glial cells are CG (gray arrows) based on their location. (D): Quantifications of Pearson’s correlation coefficient (PCC) and Mander’s Overlap Coefficient (as explained in Materials and methods) (n = 8, 5). (E–H): GMR85G01-G4 is expressed in larval gut, salivary gland (SG) and wing discs (WD), but not fat body (FB), shown by inducing UAS-Stinger in the cell nucleus (n = 3, 3, 3, 3). Scale bar: 200 μm in (E) and 100 μm in (F–H). (I, J) Time-course single-section images of wild-type (I–I′″) and elavC155QF>QUAS-prosRi tumor brains (J–J′″) with FUCCI overexpressed in BBB glia using GMR85G01-G4 (G1-phase cells: red; S-phase cells: green; and G2/M-phase cells: yellow). Time points: 48, 72, 96hALH and 24hAPF in wild-type larvae; and 48, 72, 96, and 120hALH in tumor-bearing larvae (pupate between 120-144hALH). (K, L) Single section images of elavC155QF>QUAS-prosRi tumor brains stained with Repo and the cell death marker Dcp-1 (yellow arrows) under Fed and NR conditions. (K′) and (L′) are zoomed-in images of K and L. (M) Quantification of the percentage of glial cells (Repo+) undergoing cell death (Dcp-1+ Repo+) among all glia in each brain lobe in (K, L) (n = 8, 6). Data information: ALH, after larvae hatching; APF, after pupa formation. NR: 72-120hALH; Dissection: 120hALH [file pbio.3003496.s004.tiff]

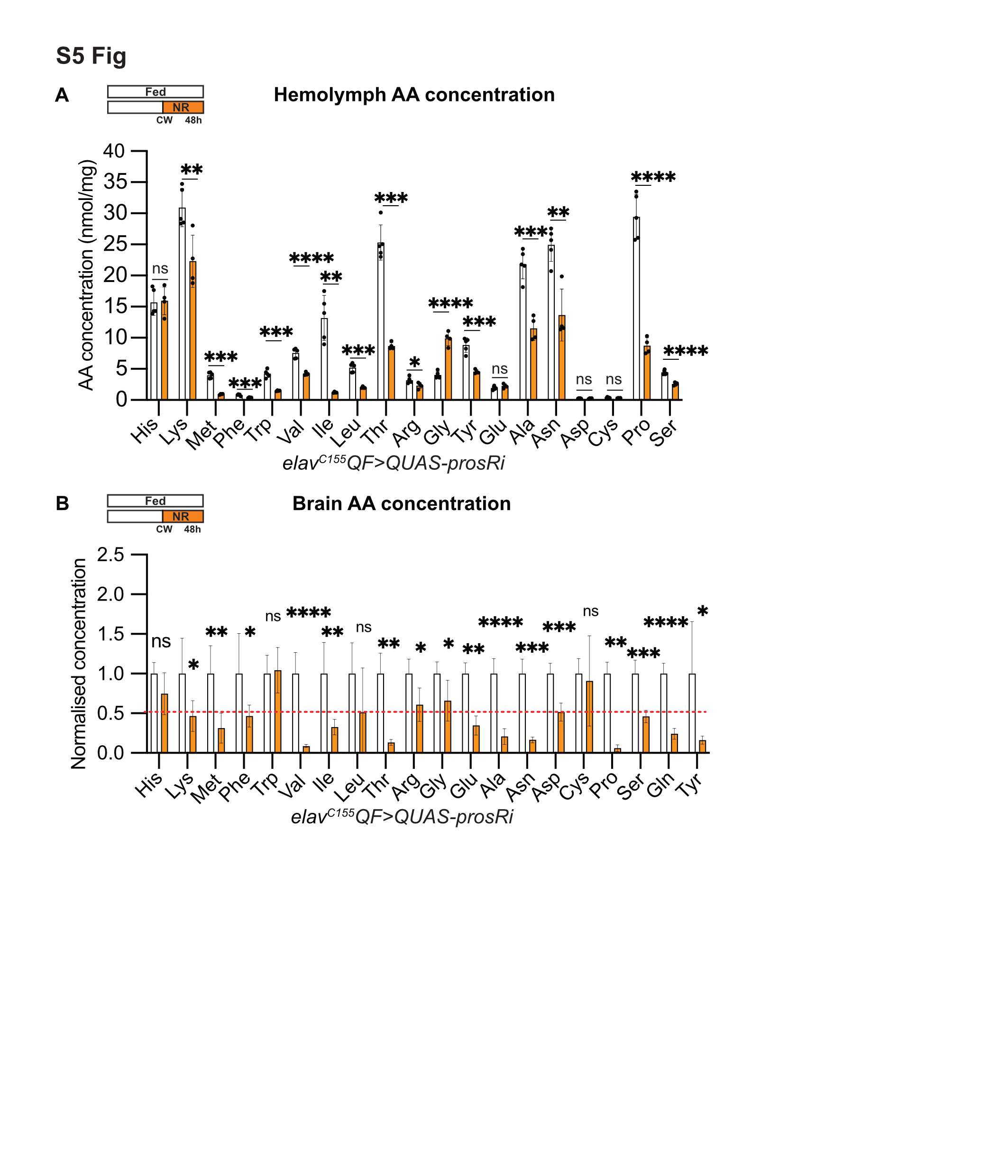

Supplement: S5 Fig — (A) Quantification of Fed versus NR hemolymph AA concentrations of elavC155QF>QUAS-prosRi tumor-bearing animals (n = 5, 4). (B) Quantifications of the normalized (to Fed) brain AA concentration in elavC155QF>QUAS-prosRi tumor-bearing animals under Fed and NR (n = 5, 5). Data information: NR: 72-120hALH; Dissection: 120hALH unless otherwise stated. Error bar represents SEM. In (A): unpaired t test, (ns) P = 0.8631; (**) P = 0.0091; (***) P = 0.0001; (***) P = 0.0002; (***) P = 0.0005; (****) P < 0.0001; (**) P = 0.0018; (***) P = 0.0004; (***) P = 0.0001; (*) P = 0.0322; (****) P < 0.0001; (***) P = 0.0002; (ns) P = 0.1710; (***) P = 0.0002; (**) P = 0.0016; (ns) P = 0.9419; (ns) P = 0.2580; (****) P < 0.0001; (****) P < 0.0001. In (B): Lys: unpaired t test, (*) P = 0.0402; Met: unpaired t test, (**) P = 0.0048; Phe: Mann–Whitney test, (*) P = 0.0317; Val: unpaired t test, (****) P < 0.0001; Ile: unpaired t test, (**) P = 0.0059; Thr: Welch’s t test, (**) P = 0.0015; Arg: unpaired t test, (*) P = 0.0138; Gly: unpaired t test, (*) P = 0.0334; Glu: Mann–Whitney test, (**) P = 0.0079; Ala: unpaired t test, (****) P < 0.0001; Asn: unpaired t test, (***) P = 0.0001; Asp: unpaired t test, (***) P = 0.0003; Pro: Mann–Whitney test, (**) P = 0.0079; Ser: unpaired t test, (***) P = 0.0002; Gln: unpaired t test, (****) P < 0.0001; Tyr: Welch’s t test, (*) P = 0.0453. Raw data are included in S3 Data. (TIFF) [file pbio.3003496.s005.tiff]

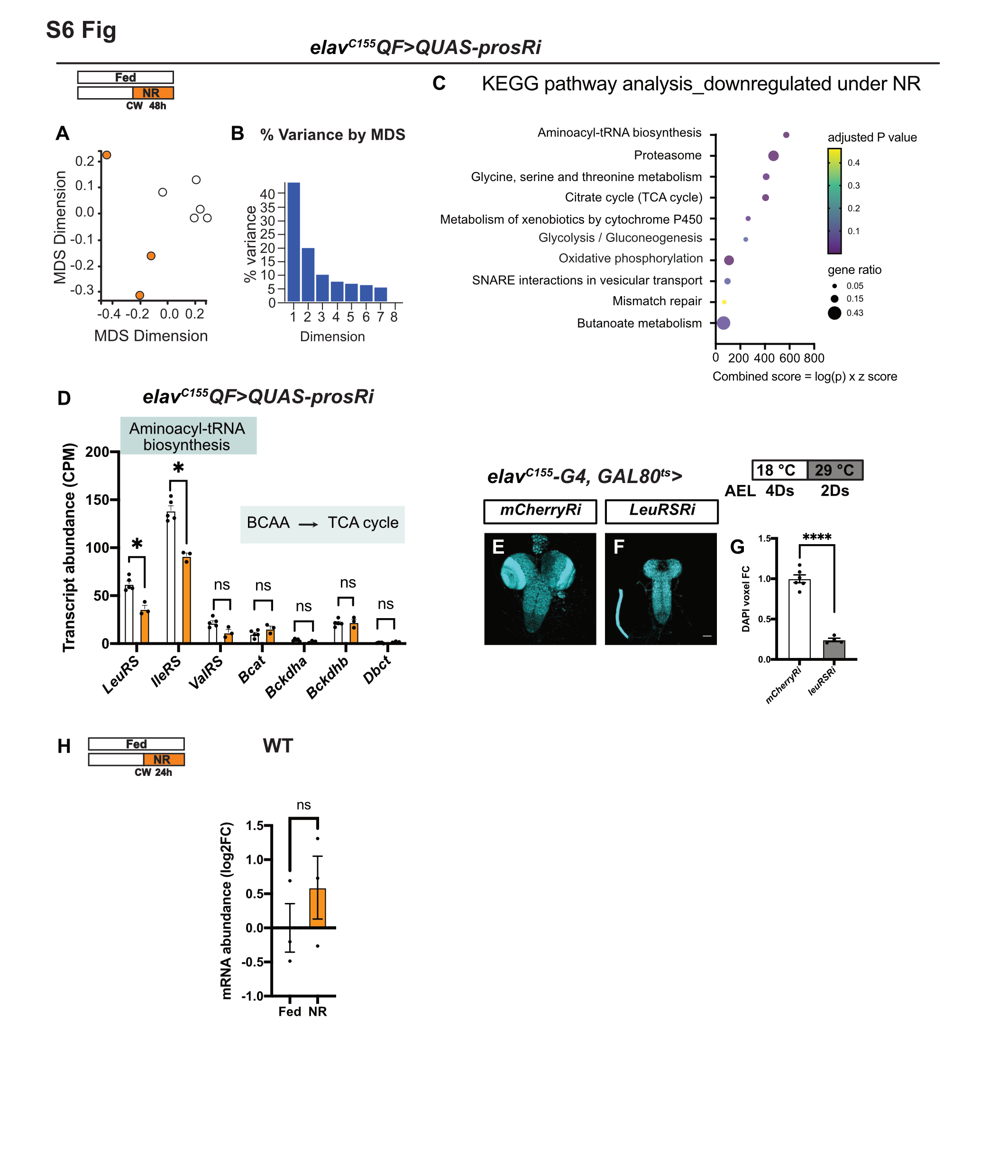

Supplement: S6 Fig — (A) Multidimensional Scaling (MDS) plot, showing gene expression profiles of elavC155QF>QUAS-prosRi brain tumor samples are separated by nutrient conditions (Fed versus NR). NR: 72-120hALH. (B) The variance table displaying how much each dimension in the MDS plot accounts for the total variance in the data. (C) KEGG pathway analysis showing top 10 downregulated pathways, ranked by the FlyEnrichr combined score. (D) Plot of transcript abundance alteration of LeuRS, IleRS, ValRS, Bcat, Bckdha, Bckdhb and Dbct in the tumor brains under NR versus Fed (from RNA seq data, n = 5, 3). (E, F) Maximum projection images of wild-type brains, where mCherryRi or LeuRSRi was overexpressed in NBs using elavC155-G4,GAL80ts. Larvae were raised at 18 °C for 4 days before being moved to 29 °C for transgene activation (2 days). Brains are stained with DAPI. (G) Quantifications of the normalized (to mCherryRi) DAPI voxels of each brain in (E and F) (n = 6, 4). (H) Quantification of mRNA level of LeuRS in wild-type brains under NR versus fed (by RT-qPCR, n = 3,3). NR: 63.5-96hALH. Data information: NR: 72–120 hALH; Dissection: 120hALH unless otherwise stated. Error bar represents SEM. In (D): (*) FDR = 0.024; (*) FDR = 0.043; (ns) FDR > 0.05. In (G): unpaired t test, (****) P < 0.0001. In (H): unpaired t test, (ns) P = 0.3666. Raw data are included in S3 Data. (TIFF) [file pbio.3003496.s006.tiff]

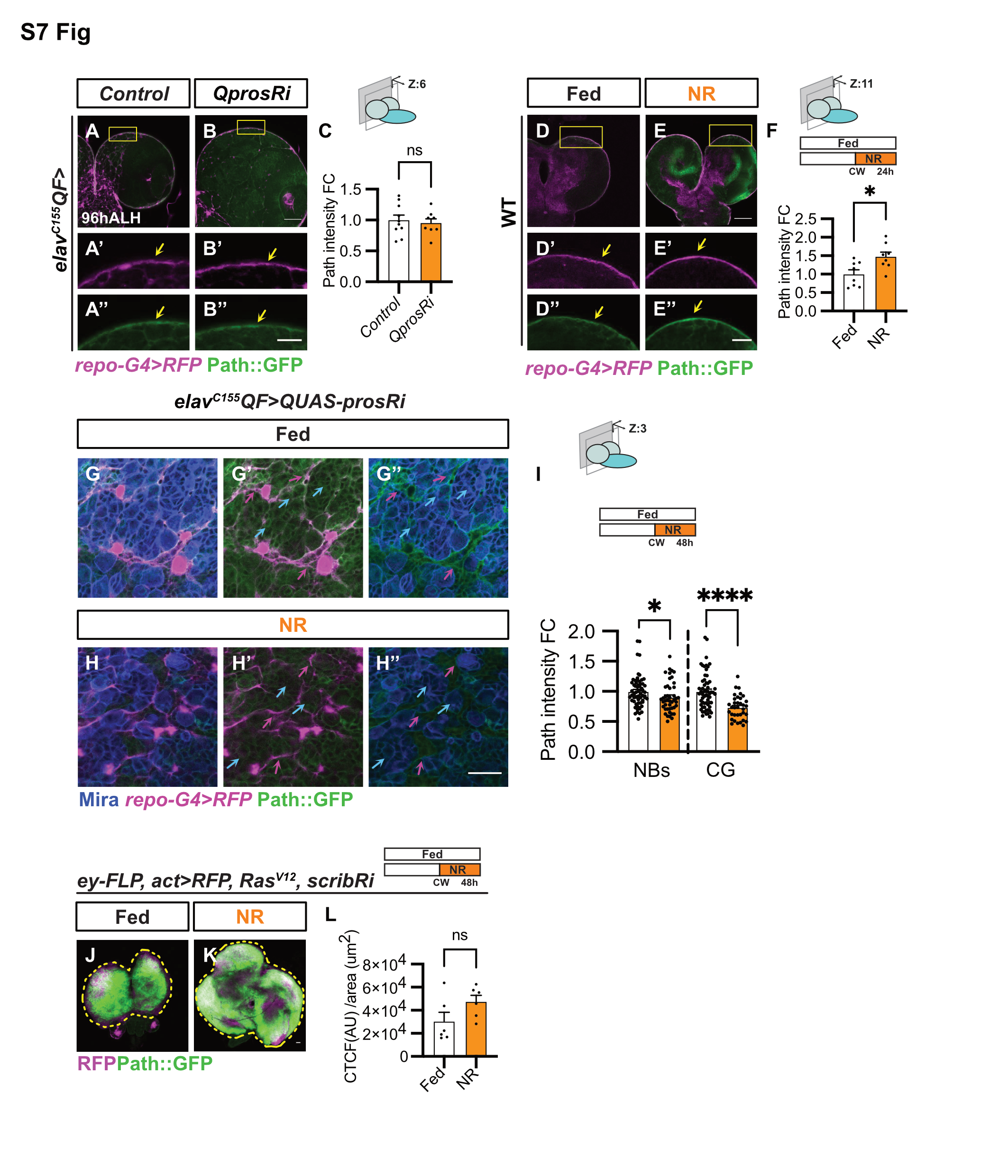

Supplement: S7 Fig — (A–B″) Single-section (A and B) and zoomed-in images (A′–B″) of Path-GFP expression in control versus elavC155QF>QUAS-prosRi tumor brains at 96hALH. Glia at the brain surface are marked with repo-G4>mRFP and distinguished from the other glial types based on position (yellow arrows). Scale bar = 20 μm in (A′–B″). (C) Quantification of the normalized (to control) Path-GFP intensity at the BBB in (A′ and B′) (n = 10, 8). (D–E) Single-section (D and E) and zoomed-in images (D′–E″) of Path-GFP expression in wild-type brains under Fed and NR. Glial membrane at the brain surface is marked with repo-G4>mRFP (yellow arrows). NR: 65 -96hALH; Dissection: 96hALH. Scale bar = 20 μm in (D′–E″). (F) Quantification of the normalized (to Fed) Path-GFP intensity at the BBB in (D and E) (n = 8, 8). (G–H″) Single-section images of Path-GFP expression in NBs (Mira, blue arrows) and CG (repo-G4>RFP, magenta arrows, distinguished from other glia based on location) in the elavC155QF>QUAS-prosRi tumor brains under Fed and NR. Scale bar = 20 μm. (I) Quantification of the normalized (to Fed) Path-GFP intensity of NBs and CG in (G–H″) (n = 63, 45, 60, 35). (J, K) Maximum projection images of Path-GFP expression in ey-FLP, act-G4>RasV12, scribRi eye disc tumors, marked by UAS-RFP under Fed and NR. (L) Quantification of Path-GFP intensity (CTCF, described in Materials and methods) of circled tumor normalized to the tumor area in (J, K) (n = 6, 6). (J–L) are from the same experiment as Fig 1W–1Y. Data information: ALH = hours after larvae hatching. NR: 72-120hALH; Dissection: 120hALH unless otherwise stated. Brain lobes are circled with yellow dashed lines. Scale bar = 50 μm. Error bar represents SEM. In (C): unpaired t test, (ns) P = 0.6740. In (F): unpaired t test, (*) P = 0.0144. In (I): Mann–Whitney test, (*) P = 0.0240; Mann–Whitney test, (****) P < 0.0001. In (L): Mann–Whitney test, (ns) P = 0.1320. Raw data are included in S3 Data. (TIFF) [file pbio.3003496.s007.tiff]

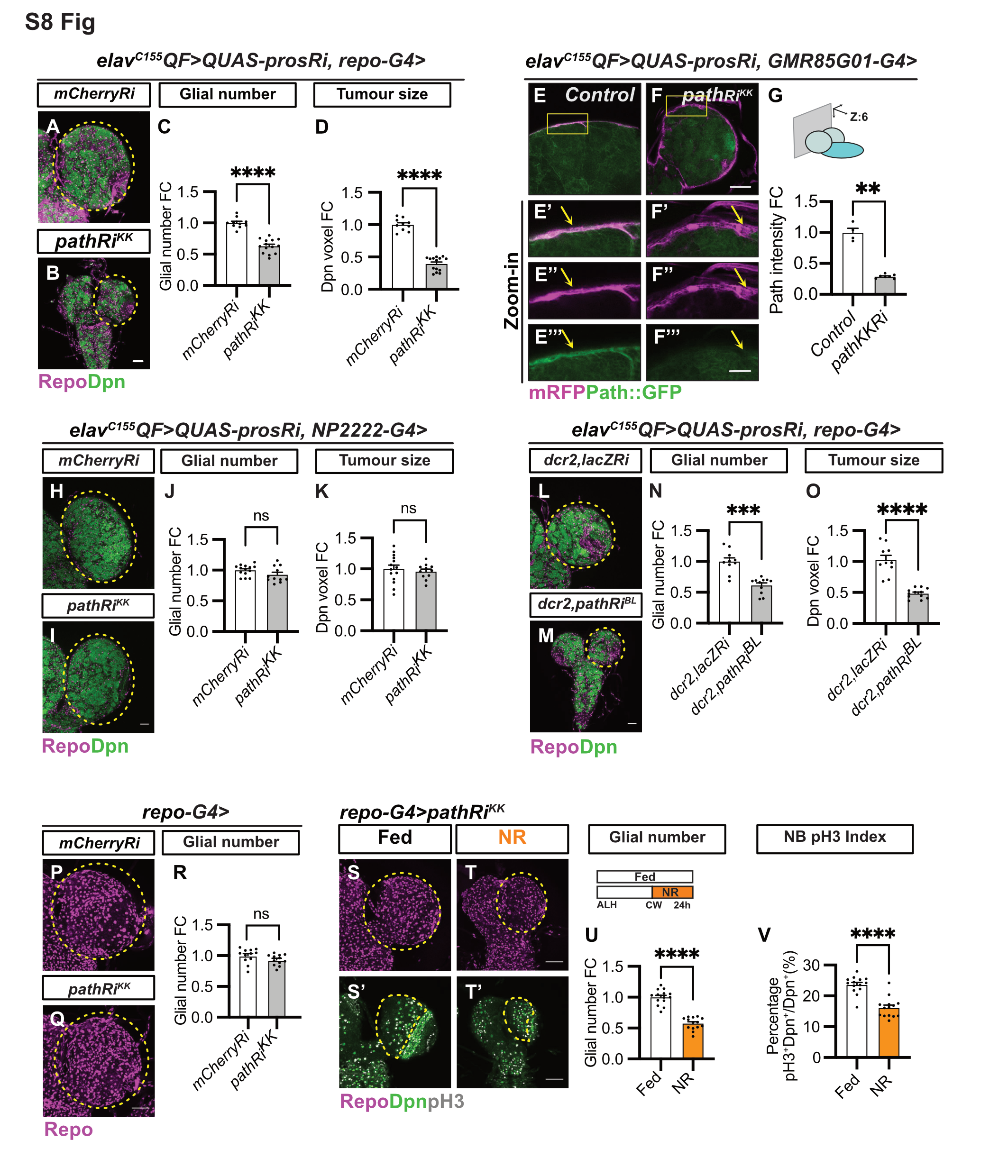

Supplement: S8 Fig — (A, B) Single section images of elavC155QF>QUAS-prosRi tumor brains, where mCherryRi and pathRiKK were overexpressed in glia using repo-G4. Glia: Repo; NBs: Dpn. (C, D) Quantifications of the normalized (to mCherryRi) glial number (C) and Dpn voxels (D) of each circled brain lobe in (A, B) (n = 10, 14). (E–F) Single-section (E and F) and zoomed-in images (E′–F′″) of Path-GFP expression (yellow arrows) in elavC155QF>QUAS-prosRi tumor brains, where pathRiKK was overexpressed in BBB glia using GMR85G01-G4, compared with control (CyOYFP siblings) at 96hALH. Scale bar = 20 μm in (E′–F′″). (G) Quantification of the normalized (to control) Path-GFP intensity at the BBB in (E–F) (n = 4, 7). (H, I) Single section images of elavC155QF>QUAS-prosRi tumor brains with pathRiKK (I) overexpressed specifically in CG using NP2222-G4, compared to mCherryRi (H) at 120hALH. Glia: Repo; NBs: Dpn. (J, K) Quantifications of the normalized (to mCherryRi) glial number (J) and Dpn voxels (K) of each circled brain lobe in (H, I) (n = 14, 11). (L, M) Single section images of elavC155QF>QUAS-prosRi tumor brains with an independent pathRi co-overexpressed with dcr2 (M) in glia using repo-G4, compared to dcr2, lacZRi (L) at 120hALH. Glia: Repo; NBs: Dpn. (N, O) Quantifications of the normalized (to dcr2, lacZRi) glial number (N) and Dpn voxels (O) of each circled brain lobe in (L, M) (n = 10, 11). (P, Q) Maximum projection images of wild-type brain lobes with pathRiKK (Q) overexpressed in glia using repo-G4, compared to mCherryRi (P) at 96hALH. Glia: Repo. (R) Quantifications of the normalized (to mCherryRi) glial number of each circled brain lobe in (P, Q) (n = 12, 12). (S–T′) Wild-type brain lobes where pathRiKK was overexpressed in glia using repo-G4 under Fed and NR. Glia: Repo (S and T, maximum projection), Dpn and pH3 in (S′ and T′, single sections). CBs are circled by yellow dashed lines. NR: 65-89hALH; Dissection: 89hALH. (U) Quantification of the normalized (to Fed) glial number of each c [file pbio.3003496.s008.tiff]

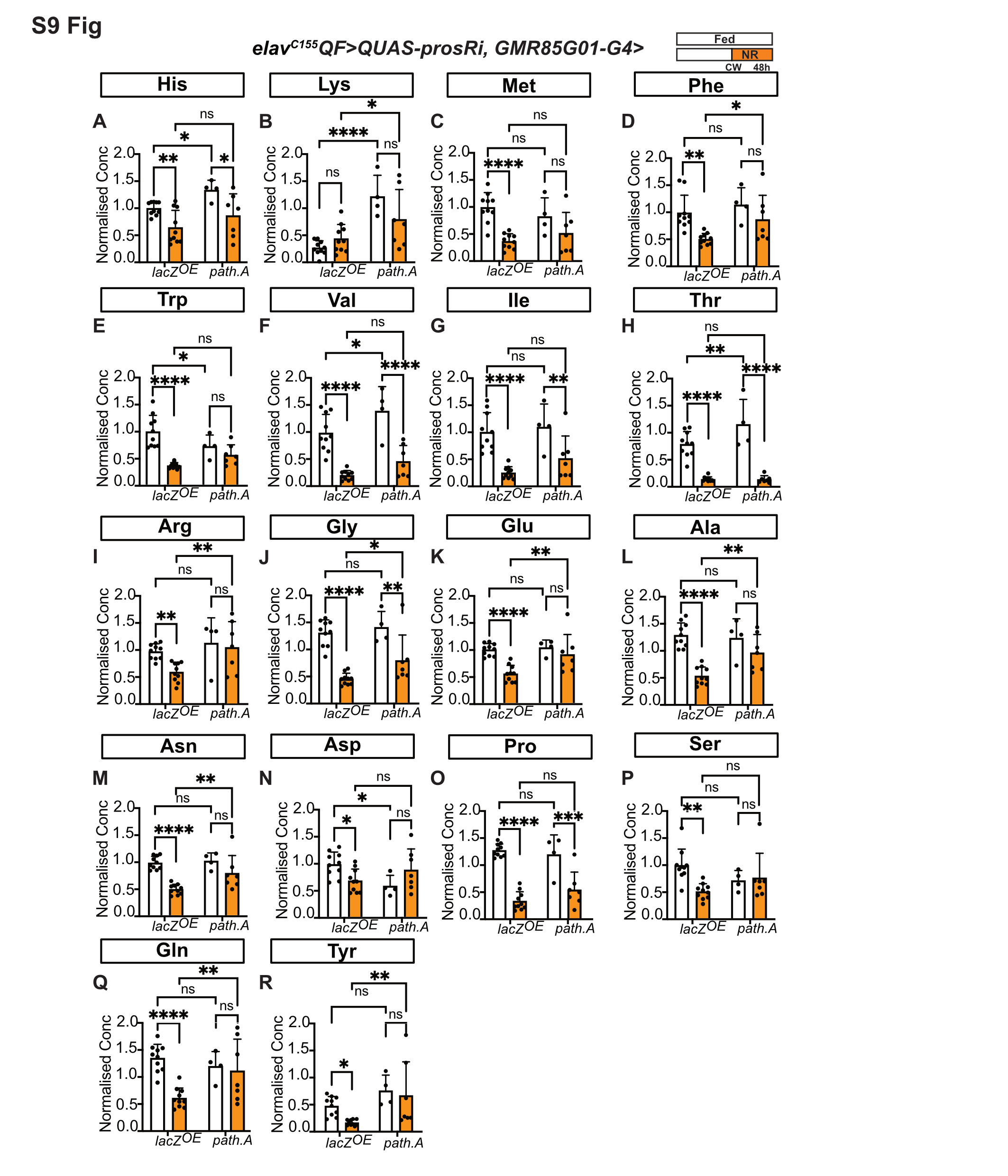

Supplement: S9 Fig — (A–R) Quantifications of the normalized (to lacZOE_Fed) brain AA concentration in elavC155QF>QUAS-prosRi tumor-bearing animals, where lacZ or path is overexpressed using GMR85G01-G4 under Fed and -Yeast conditions (n = 10, 10, 4, 7). Data information: Two-way ANOVA were used to analyze whether the effect of yeast dropout on brain AA concentration is restored upon Path overexpression in the BBB. Statistical results including multiple comparisons are displayed in S1M Table. Raw data are included in S3 Data. (TIFF) [file pbio.3003496.s009.tiff]

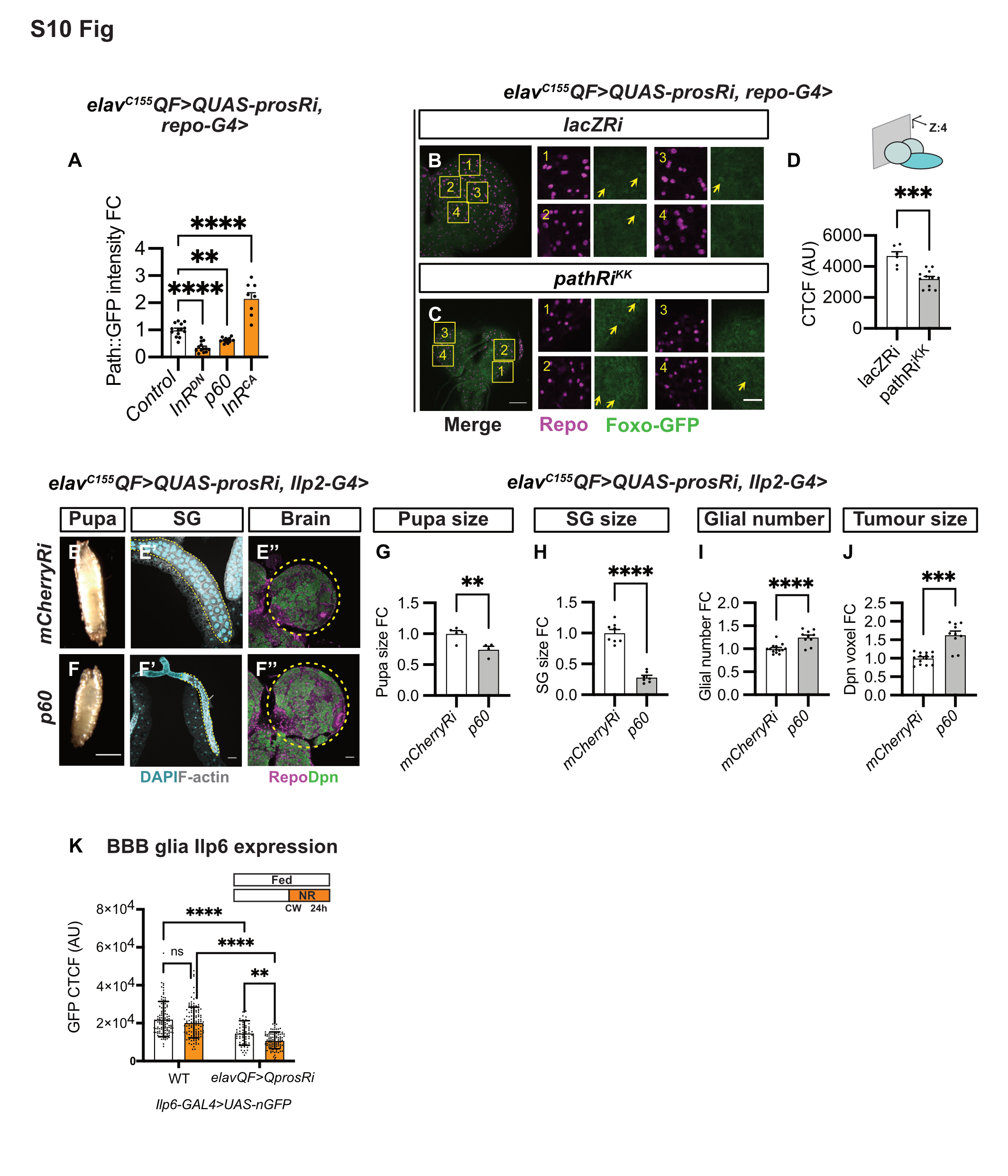

Supplement: S10 Fig — (A) Quantification of Path-GFP expression at the surface glia of the elavC155QF>QUAS-prosRi tumor brains with InRDN, p60 or InRCA overexpressed in glia using repo-G4 at 120hALH (n = 14, 13, 12, 8). (B, C) surface-section images and zoomed-in images of Foxo-GFP expression (Yellow arrows) in elavC155QF>QUAS-prosRi tumor brains, where lacZRi or pathRiKK was overexpressed using repo-G4. Glia: Repo. Dissection: 96hALH. Scale bar = 50 μm in B and C. Scale bar = 20 μm in zoomed-in images (1–4). (D) Quantification of glial nuclear Foxo-GFP intensity in (B, C) (n = 6, 12). (E, F) Pupa, salivary glands (SG, stained with Phalloidin and DAPI) and brains (stained with Repo and Dpn) in elavC155QF>QUAS-prosRi tumor-bearing animals, where p60 was overexpressed in insulin-producing cells (IPCs) using Ilp2-G4, compared with mCherryRi at 120hALH. (E′) and (F′): maximum projection images; (E″) and (F″): single section images; Scale bar = 1 mm in (E and F). (G) Quantification of the normalized (to mCherryRi) pupal size (area) in (E and F) (n = 5, 4). (H) Quantification of the normalized (to mCherryRi) SG size (area) in (E′ and F′) (n = 8, 7). (I, J) Quantifications of the normalized (to mCherryRi) glial number (I) and Dpn voxels (J) of each circled brain lobe in (E″ and F″) (n = 14, 10). (K) Quantifications of the GFP intensity (CTCF) in BBB glial nucleus in wild-type and elavC155QF>QUAS-prosRi tumor brains under Fed and NR conditions. Wild-type and tumor-bearing animals were starved after CW (65hALH and 68hALH, respectively) (n = 120, 120, 60, 100 cells from 12, 12, 6, 10 brain lobes). Data information: ALH = after larvae hatching. Brain lobes are circled with yellow dashed lines. Scale bar = 50 μm. Error bar represents SEM. In (A): One-way ANOVA, (****) P < 0.0001, (**) P = 0.0073, (****) P < 0.0001. In (D): unpaired t test, (***) P = 0.0001. In (G): unpaired t test, (**) P = 0.0098. In (H): unpaired t test, (****) P < 0.0001. In (I): unpaired t test, (****) P < 0.0001. In (J): Welch’ [file pbio.3003496.s010.tiff]
