# Supplementary material for: The blood–brain barrier regulates brain tumor growth through the SLC36 amino acid transporter Pathetic in Drosophila
Source: PLoS Biol. 2025 Nov 18;23(11):e3003496. doi: 10.1371/journal.pbio.3003496 (PMC12626262; doi:10.1371/journal.pbio.3003496)
Supplement: S1 Table — (DOCX) [file pbio.3003496.s014.docx]

**S1 Table**

**A**

| **Fig 1H. Data and statistics** | | | | | |
| --- | --- | --- | --- | --- | --- |
| Mean ± SEM | | | | | |
| Genotype | *w1118* | | | *elavQF>QUAS-prosRi* | |
| Fed | 1.00±0.04 | | | 8.08±0.30 | |
| NR | 0.98±0.02 | | | 6.54±0.19 | |
| Two-way ANOVA interaction and multiple comparisons | | | | | |
| Comparisons | *w1118_*  Fed vs NR | *elavQF>QUAS-prosRi*  Fed vs NR | *w1118_*Fed  vs *elavQF>QUAS-prosRi* *_*Fed | | *w1118_*NR  vs *elavQF>QUAS-prosRi* *_*NR |
| Adjusted P Value | ns 0.9995 | **** <0.0001 | **** <0.0001 | | **** <0.0001 |
| Interaction P value | **** <0.0001 | | | | |

**B**

| **Fig 1I. Data and statistics** | | | | | |
| --- | --- | --- | --- | --- | --- |
| Mean ± SEM | | | | | |
| Genotype | *w1118* | | | *elavQF>QUAS-prosRi* | |
| Fed | 4.67±0.26 | | | 8.39±0.39 | |
| NR | 4.68±0.40 | | | 6.57±0.38 | |
| Two-way ANOVA interaction and multiple comparisons | | | | | |
| Comparisons | *w1118_*  Fed vs NR | *elavQF>QUAS-prosRi*  Fed vs NR | *w1118_*Fed  vs *elavQF>QUAS-prosRi* *_*Fed | | *w1118_*NR  vs *elavQF>QUAS-prosRi* *_*NR |
| Adjusted P Value | ns >0.9999 | ** p =0.002 | **** <0.0001 | | ** p =0.003 |
| Interaction P value | * p=0.0138 | | | | |

**C**

| **Fig 2G. Data and statistics** | | | | | |
| --- | --- | --- | --- | --- | --- |
| Mean ± SEM | | | | | |
| Genotype | *w1118* | | | *elavQF>QUAS-prosRi* | |
| Fed | 1.00±0.04 | | | 2.01±0.13 | |
| NR | 0.87±0.05 | | | 1.62±0.09 | |
| Two-way ANOVA interaction and multiple comparisons | | | | | |
| Comparisons | *w1118_*  Fed vs NR | *elavQF>QUAS-prosRi*  Fed vs NR | *w1118_*Fed  vs *elavQF>QUAS-prosRi* *_*Fed | | *w1118_*NR  vs *elavQF>QUAS-prosRi* *_*NR |
| Adjusted P Value | ns 0.6227 | * p =0.0013 | **** <0.0001 | | **** <0.0001 |
| Interaction P value | ns p=0.1270 | | | | |

**D**

| **Fig 2J. Data and statistics** | | | |
| --- | --- | --- | --- |
| Mean ± SEM | | | |
|  | G1 | S | G2/M |
| 48hALH | 16.98±1.49 | 41.16±3.10 | 41.86±2.40 |
| 72hALH | 48.52±1.37 | 32.06±0.94 | 19.42±1.01 |
| 96hALH | 32.02±2.93 | 34.24±1.66 | 33.74±3.95 |
| 120hALH/24hAPF | 36.21±2.25 | 16.79±2.08 | 47.00±1.95 |
| Two-way ANOVA interaction and multiple comparisons | | | |
| Adjusted P Value | 48-72ALH | 72-96ALH | 96-120ALH/24APF |
| G1 | **** <0.0001 | ***0.0006 | ns 0.9965 |
| S | ns 0.1847 | ns > 0.9999 | ** 0.0030 |
| G2/M | **** <0.0001 | ** 0.0051 | ns 0.0687 |
| Interaction P value | **** <0.0001 | | |

**E**

| **Fig 2K. Data and statistics** | | | |
| --- | --- | --- | --- |
| Mean ± SEM | | | |
|  | G1 | S | G2/M |
| 48hALH | 9.91±1.30 | 23.85±3.67 | 66.24±4.64 |
| 72hALH | 41.75±4.42 | 39.54±1.79 | 18.71±2.76 |
| 96hALH | 48.23±1.94 | 33.66±1.63 | 18.12±1.17 |
| 120hALH | 51.75±2.12 | 24.22±2.00 | 24.03±1.54 |
| Two-way ANOVA interaction and multiple comparisons | | | |
| Adjusted P Value | 48-72ALH | 72-96ALH | 96-120ALH |
| G1 | **** <0.0001 | ns 0.9429 | ns 0.9998 |
| S | * 0.0179 | ns 0.9708 | ns 0.6878 |
| G2/M | **** <0.0001 | ns > 0.9999 | ns 0.9812 |
| Interaction P value | **** <0.0001 | | |

**F**

| **Fig 2M. Data and statistics** | | | |
| --- | --- | --- | --- |
| Mean ± SEM | | | |
|  | G1 | S | G2/M |
| Fed | 46.97±1.43 | 22.25±1.15 | 30.78±1.91 |
| NR | 48.10±1.77 | 12.18±0.93 | 39.72±1.33 |
| Two-way ANOVA interaction and multiple comparisons | | | |
| Adjusted P Value | G1 | S | G2/M |
| Fed-NR | ns 0.9320 | **** <0.0001 | *** 0.0005 |
| Interaction P value | **** <0.0001 | | |
| Chi-square test | | | |
| Fed-NR | ****** 0.0014 | | |

**G**

| **Fig 2U. Data and statistics** | | | | | |
| --- | --- | --- | --- | --- | --- |
| Mean ± SEM | | | | | |
| Genotype | *mCherryRi*_ | | | *Cdk4CycD^OE^* | |
| Fed | 1.00±0.02 | | | 1.48±0.04 | |
| NR | 0.52±0.06 | | | 1.18±0.05 | |
| Two-way ANOVA interaction and multiple comparisons | | | | | |
| Comparisons | *mCherryRi_*  Fed vs NR | *Cdk4CycD^OE^*  Fed vs NR | *mCherryRi_*Fed  vs *Cdk4CycD^OE^_*Fed | | *mCherryRi_*NR  vs *Cdk4CycD^OE^_*NR |
| Adjusted P Value | **** <0.0001 | *** 0.0002 | **** <0.0001 | | **** <0.0001 |
| Interaction P value | * 0.0446 | | | | |

**H**

| **Fig 2V. Data and statistics** | | | | | |
| --- | --- | --- | --- | --- | --- |
| Mean ± SEM | | | | | |
| Genotype | *mCherryRi*_ | | | *Cdk4CycD^OE^* | |
| Fed | 1.00±0.03 | | | 1.53±0.04 | |
| NR | 0.37±0.03 | | | 1.25±0.09 | |
| Two-way ANOVA interaction and multiple comparisons | | | | | |
| Comparisons | *mCherryRi_*  Fed vs NR | *Cdk4CycD^OE^*  Fed vs NR | *mCherryRi_*Fed  vs *Cdk4CycD^OE^_*Fed | | *mCherryRi_*NR  vs *Cdk4CycD^OE^_*NR |
| Adjusted P Value | **** <0.0001 | ** 0.0017 | **** <0.0001 | | **** <0.0001 |
| Interaction P value | ** 0.0013 | | | | |

**I**

| **Fig 3O. Data and statistics** | | | |
| --- | --- | --- | --- |
| Mean ± SEM | | | |
|  | G1 | S | G2/M |
| CDD | 25.92±1.56 | 43.66±2.00 | 30.42±1.76 |
| CDD-Leu | 46.52±3.74 | 25.88±2.40 | 27.60±2.03 |
| CDD-Ile | 37.01±3.03 | 31.68±2.48 | 31.32±2.23 |
| CDD-Met | 25.00±3.30 | 45.21±4.00 | 29.81±2.73 |
| Two-way ANOVA multiple comparisons | | | |
| Adjusted P Value | CDD-Leu vs CDD | CDD-Ile vs CDD | CDD-Met vs CDD |
| G1 | **** <0.0001 | ** 0.0090 | ns > 0.9999 |
| S | **** <0.0001 | ** 0.0036 | ns > 0.9999 |
| G2/M | ns= 0.9956 | ns > 0.9999 | ns > 0.9999 |
| Chi-square test | **** <0.0001 | *** 0.0001 | ns 0.6442 |

**J**

| **Fig 4I. Data and statistics** | | | | | |
| --- | --- | --- | --- | --- | --- |
| Mean ± SEM | | | | | |
| Genotype | *Control_w1118* | | | *path.A* | |
| Fed | 1.00±0.05 | | | 1.03±0.05 | |
| -Y | 0.42±0.04 | | | 0.74±0.03 | |
| Two-way ANOVA interaction and multiple comparisons | | | | | |
| Comparisons | *Control_*  Fed vs -Y | *path.A_*  Fed vs -Y | *Control _*Fed  vs *path.A _*Fed | | *Control _*-Y  vs *path.A _*-Y |
| Adjusted P Value | **** <0.0001 | **** <0.0001 | ns 0.9701 | | **** <0.0001 |
| Interaction P value | ** 0.0013 | | | | |

**K**

| **Fig 4J. Data and statistics** | | | | | |
| --- | --- | --- | --- | --- | --- |
| Mean ± SEM | | | | | |
| Genotype | *Control_w1118* | | | *path.A* | |
| Fed | 1.00±0.10 | | | 1.02±0.09 | |
| -Y | 0.34±0.06 | | | 0.70±0.06 | |
| Two-way ANOVA interaction and multiple comparisons | | | | | |
| Comparisons | *Control_*  Fed vs -Y | *path.A_*  Fed vs -Y | *Control _*Fed  vs *path.A _*Fed | | *Control _*-Y  vs *path.A _*-Y |
| Adjusted P Value | **** <0.0001 | * 0.0207 | ns 0.9963 | | ** 0.0021 |
| Interaction P value | * 0.033 | | | | |

**L**

| **Fig 4N. Data and statistics** | | | | | |
| --- | --- | --- | --- | --- | --- |
| Leu: Mean ± SEM | | | | | |
| Genotype | *Control_lacZ^OE^* | | | *path.A* | |
| Fed | 1.00±0.10 | | | 1.09±0.20 | |
| -Y | 0.30±0.04 | | | 0.71±0.20 | |
| Two-way ANOVA interaction and multiple comparisons | | | | | |
| Comparisons | *lacZ^OE^ _*  Fed vs -Y | *path.A_*  Fed vs -Y | *lacZ^OE^ _*Fed  vs *path.A _*Fed | | *lacZ^OE^ _*-Y  vs *path.A _*-Y |
| Adjusted P Value | **** <0.0001 | ns 0.0832 | ns 0.6666 | | * 0.023 |

**M**

| **S9 Fig. Data and statistics** | | | | |
| --- | --- | --- | --- | --- |
| Two-way ANOVA interaction and multiple comparisons | | | | |
| Adjusted P Value | *lacZ^OE^ _*  Fed vs -Y | *path.A_*  Fed vs -Y | *lacZ^OE^ _*Fed  vs *path.A _*Fed | *lacZ^OE^ _*-Y  vs *path.A _*-Y |
| His | ** 0.0082 | * 0.0114 | * 0.0493 | ns 0.1204 |
| Lys | ns 0.2601 | ns 0.0542 | ****<0.0001 | * 0.0409 |
| Met | ****<0.0001 | ns 0.0870 | ns 0.3125 | ns 0.2827 |
| Phe | ** 0.0013 | ns 0.1680 | ns 0.4205 | * 0.0219 |
| Trp | ****<0.0001 | ns 0.2280 | * 0.0359 | ns 0.0668 |
| Val | ****<0.0001 | ****<0.0001 | * 0.0249 | ns 0.0793 |
| Ile | ****<0.0001 | ** 0.0083 | ns 0.6264 | ns 0.1086 |
| Thr | ****<0.0001 | ****<0.0001 | ** 0.0060 | ns 0.9751 |
| Arg | ** 0.0092 | ns 0.6808 | ns 0.3913 | ** 0.0049 |
| Gly | ****<0.0001 | ** 0.0020 | ns 0.5507 | * 0.0210 |
| Glu | ****<0.0001 | ns 0.3443 | ns 0.7530 | ** 0.0019 |
| Ala | ****<0.0001 | ns 0.1032 | ns 0.7273 | ** 0.0020 |
| Asn | ****<0.0001 | ns 0.0579 | ns 0.7453 | ** 0.0026 |
| Asp | * 0.0168 | ns 0.0843 | * 0.0170 | ns 0.1357 |
| Pro | ****<0.0001 | *** 0.0001 | ns 0.5751 | ns 0.0763 |
| Ser | ** 0.0010 | ns 0.7848 | ns 0.1188 | ns 0.0894 |
| Gln | ****<0.0001 | ns 0.6999 | ns 0.4626 | ** 0.0057 |
| Tyr | * 0.0439 | ns 0.6710 | ns 0.1558 | ** 0.0042 |

**N**

| **Fig 5T. Data and statistics** | | | | | |
| --- | --- | --- | --- | --- | --- |
| Mean ± SEM | | | | | |
| Genotype | *mCherryRi*_ | | | *myrAKT^OE^* | |
| Fed | 1.00±0.04 | | | 1.10±0.07 | |
| NR | 0.27±0.02 | | | 0.82±0.09 | |
| Two-way ANOVA interaction and multiple comparisons | | | | | |
| Comparisons | *mCherryRi_*  Fed vs NR | *myrAKT^OE^*  Fed vs NR | *mCherryRi_*Fed  vs *myrAKT^OE^_*Fed | | *mCherryRi_*NR  vs *myrAKT^OE^_*NR |
| Adjusted P Value | **** <0.0001 | * 0.013 | ns 0.5646 | | **** <0.0001 |
| Interaction P value | *** 0.0002 | | | | |

**O**

| **Fig 5U. Data and statistics** | | | | | |
| --- | --- | --- | --- | --- | --- |
| Mean ± SEM | | | | | |
| Genotype | *mCherryRi*_ | | | *myrAKT^OE^* | |
| Fed | 1.00±0.04 | | | 1.16±0.07 | |
| NR | 0.23±0.02 | | | 0.87±0.08 | |
| Two-way ANOVA interaction and multiple comparisons | | | | | |
| Comparisons | *mCherryRi_*  Fed vs NR | *myrAKT^OE^*  Fed vs NR | *mCherryRi_*Fed  vs *myrAKT^OE^_*Fed | | *mCherryRi_*NR  vs *myrAKT^OE^_*NR |
| Adjusted P Value | **** <0.0001 | * 0.0112 | ns 0.2098 | | **** <0.0001 |
| Interaction P value | *** 0.0001 | | | | |

**P**

| **Fig 7I. Data and statistics** | | | | | | |
| --- | --- | --- | --- | --- | --- | --- |
| Mean ± SEM | | | | | | |
| Genotype | *mCherryRi*_ | | *Rag^CA^* | | *S6K^CA^* | |
| Fed | 1.00±0.04 | | 0.96±0.06 | | 0.85±0.03 | |
| NR | 0.46±0.02 | | 0.77±0.02 | | 0.53±0.03 | |
| Two-way ANOVA interaction and multiple comparisons (*mCherryRi vs Rag^CA^*) | | | | | | |
| Comparisons | *mCherryRi_*  Fed vs NR | *Rag^CA^* _Fed vs NR | | *mCherryRi_*Fed  vs *Rag^CA^_*Fed | | *mCherryRi_*NR  vs *Rag^CA^_*NR |
| Adjusted P Value | **** <0.0001 | ** 0.0014 | | ns 0.8934 | | **** <0.0001 |
| Interaction P value | **** <0.0001 | | | | | |
| Two-way ANOVA interaction and multiple comparisons (*mCherryRi vs S6K^CA^*) | | | | | | |
| Comparisons | *mCherryRi_*  Fed vs NR | *S6K^CA^* _Fed vs NR | | *mCherryRi_*Fed  vs *S6K^CA^_*Fed | | *mCherryRi_*NR  vs *S6K^CA^_*NR |
| Adjusted P Value | **** <0.0001 | **** <0.0001 | | * 0.0111 | | ns 0.3616 |
| Interaction P value | **0.0012 | | | | | |

**Q**

| **Fig 7J. Data and statistics** | | | | | | |
| --- | --- | --- | --- | --- | --- | --- |
| Mean ± SEM | | | | | | |
| Genotype | *mCherryRi*_ | | *Rag^CA^* | | *S6K^CA^* | |
| Fed | 1.00±0.04 | | 1.03±0.11 | | 0.71±0.03 | |
| NR | 0.35±0.02 | | 0.72±0.03 | | 0.45±0.02 | |
| Two-way ANOVA interaction and multiple comparisons (*mCherryRi vs Rag^CA^*) | | | | | | |
| Comparisons | *mCherryRi_*  Fed vs NR | *Rag^CA^* _Fed vs NR | | *mCherryRi_*Fed  vs *Rag^CA^_*Fed | | *mCherryRi_*NR  vs *Rag^CA^_*NR |
| Adjusted P Value | **** <0.0001 | ** 0.0013 | | ns 0.9882 | | **** <0.0001 |
| Interaction P value | ** <0.0038 | | | | | |
| Two-way ANOVA interaction and multiple comparisons (*mCherryRi vs S6K^CA^*) | | | | | | |
| Comparisons | *mCherryRi_*  Fed vs NR | *S6K^CA^* _Fed vs NR | | *mCherryRi_*Fed  vs *S6K^CA^_*Fed | | *mCherryRi_*NR  vs *S6K^CA^_*NR |
| Adjusted P Value | **** <0.0001 | **** <0.0001 | | **** <0.0001 | | ns 0.1189 |
| Interaction P value | **** <0.0001 | | | | | |

**R**

| Medium | Composition |
| --- | --- |
| Standard food | 5.25% frozen yeast, 4.38% glucose, 4.97% Polenta and 0.42% Agar |
| NR | 0.42% Agar/ PBS |
| -Yeast | 4.38% glucose, 4.97% Polenta and 0.42% Agar |
| -Carbohydrate | 5.25% frozen yeast and 0.42% Agar |
| +2xYeast | 15.75% frozen yeast, 4.38% glucose, 4.97% Polenta and 0.42% Agar |
| +2xGlucose | 5.25% frozen yeast, 13.14% glucose, 4.97% Polenta and 0.42% Agar |
| CDD, CDD-Leu, CDD-Ile and CDD-Met | Prepared according to *Kosakamoto et al, 2022* |
