# Supplementary material for: The blood–brain barrier regulates brain tumor growth through the SLC36 amino acid transporter Pathetic in Drosophila
Source: PLoS Biol. 2025 Nov 18;23(11):e3003496. doi: 10.1371/journal.pbio.3003496 (PMC12626262; doi:10.1371/journal.pbio.3003496)
Supplement: Attachment — Submitted filename: Response to reviewers.docx [file pbio.3003496.s015.docx]

**ESSENTIAL:**

1- The evidence suggesting that Path levels correlate with brain BCAA levels could be strengthened. In Figure 4, you show that glial-specific knockdown of path under nutrient restriction reduces tumor growth and brain amino acid levels—particularly BCAAs—without affecting known Path substrates, suggesting a broader role for Path in regulating amino acid availability. While path knockdown (KD) was assessed for its effect on brain amino acid concentrations, no equivalent analysis was performed in the context of Path overexpression, even though it is shown that Path overexpression in PG cells partially rescues tumor growth under NR conditions (Fig. 4I-J). It remains unclear whether the observed phenotypic rescue correlates with restored levels of BCAAs.

We have measured brain AA levels under the conditions where we overexpressed Path in the BBB glia of tumour brains under fed vs -Yeast conditions. We found that Path overexpression significantly increased brain Leucine level compared to control under yeast dropout conditions (Fig 4N). The amount of other BCAAs (Val and Ile) was also increased, however, the data did not reach significance (S9F and G Fig). Furthermore, Met levels did not appear to be increased (Figure S9C). In addition, we observed rescue of many other AAs including Phe, Arg, Gly, Glu, Asn, Gln and Tyr (S9D, I-M, and Q-R Fig). Among these AAs, Asn and Tyr were reduced under Path knockdown. Together, our data suggests that Path regulates the transport of BCAAs and other AAs such as Asn and Tyr. This data is incorporated into the result part subtitled “Path regulates the availability of amino acids in the brain”.

2- The Fly-FUCCI system driven by the PG-specific GMR85G01-GAL4 driver is used to monitor cell cycle dynamics in perineural glia (PG) under fed and NR conditions. You report a significant increase in G1-phase cells and a corresponding decrease in S-phase cells under NR, suggesting a slowdown or arrest of the cell cycle. You also state that this change is not due to increased cell death, thereby implying true cell cycle arrest. However, the FUCCI system alone is insufficient to conclusively demonstrate cell cycle arrest. FUCCI provides a proxy for cell cycle phase distribution but does not differentiate between transient delay and true checkpoint-mediated arrest. To substantiate the claim of cell cycle arrest, you should perform additional experiments such as analyzing the expression of phase-specific markers (e.g., Cyclins) and examining well-established cell cycle arrest or checkpoint markers, including p21, p27, phosphorylated Chk1/Chk2, or γH2AX in the case of DNA damage-induced arrest.

We reported a significant increase in M-phase cells, and a corresponding decrease in S-phase cells under NR, suggesting either a transient delay in cell cycle progression or cell cycle arrest. To assess whether the change of the cell FUCCI profile is due to cell cycle arrest, the reviewer asks us to assess the expression of cell cycle checkpoint markers in BBB glia under Fed vs NR conditions. P21 is an inhibitor of cyclin-dependent kinases (CDKs). It can cause cell cycle arrest at G1/S and G2/M phase. We have done the staining of P21/Dacapo with BBB glial cells labelled with Stinger-GFP, and found these cells exhibited a 5-fold increase in Dacapo+ BBB glial cells in tumour brains under NR (Fig 2N-P), suggesting that cells have undergone checkpoint mediated arrest. We also tried CycB staining, we saw that there is no colocalization of CycB with BBB glia labelled with GFP, thus, the cyclins could be transient, and we could not capture the colocalised CycB within BBB glial cells. However, since we carried out FUCCI, which specifically examine multiple Cyclins, we believe our data in Fig 2 already addressed this point.

3- For statistical analysis, it seems that fed was compared to NR; however, within-group analysis (fed control compared to KD or OE) is not shown for some of the plots. This can result in misinterpretation of the results (e.g. Fig 2S-T). Based on the data, it seems that CDk4OE; CycDOE have increased glial numbers independent of fed/NR.

For Fig 2S-T, we agree that *CdK4^OE^, CycD^OE^* dramatically increased total glial numbers independent of fed/NR. This is due to its role in accelerating cell cycle progression. However, the point we are trying to convey is that the number of glia is reduced less upon *CdK4^OE^, CycD^OE^* activation (20%) compared to control (48%) under NR, which indicates the activation of *CdK4^OE^, CycD^OE^* reduced the effect of NR on glial cell number. Experimentally, we set up the four conditions (*mCherryRi* Fed vs NR, *CdK4^OE^, CycD^OE^* Fed vs NR) in parallel such that these conditions are all comparable. And statistically, two-way ANOVA was performed on raw data and was used to:

a: Assess whether the transgene activation (KD or OE) influences the effect of NR on glial number/tumour size. This question is answered by the interaction P value (specified on the graph and figure legends, e.g. P = 0.0446 in Fig 2U). A significant interaction P value indicates that the glial number is differentially affected under NR, when the transgene is overexpressed. In the example of Fig 2U, a less reduction in glial number under NR vs Fed upon *CdK4^OE^, CycD^OE^* activation indicates that glial number is more spared under NR in response to *CdK4^OE^, CycD^OE^* activation.

b. Assess the statistical significance of the changes among the four groups by multiple comparisons. We also performed this analysis; however, we only showed Fed vs NR comparisons on the graph. We have added the rest of the statistical results as requested for all group analyses to S1 Table and cited in the figure legends.

To make the point clearer, we have also added the sentence “the number of glia is reduced XX% upon NR in XX (genotype), and XX% in XX (genotype).” in the results, in all contexts where we describe group comparisons.

In summary, we conclude that there is a rescue in group analysis only when the interaction P value is significant. This analysis takes into account the fact that transgene activation itself can change glial number/tumour size even under fed conditions.

4- It is not entirely clear why the NR timelines are different between figures and even between WT and tumor (e.g. Fig 2 & 4 NR was from 72-96h while in Fig 6. The WT was NR from 65h to 92h while tumor was from 68h to 92h). Fig 3, the =Y or -GP was fed for much longer (48-120h). It is difficult to make comparisons among the groups this way.

In our experiments, we used two NR strategies:

a. We subject the animals to NR for 24 hours post CW, when we compare the effects of NR on brain growth in wildtype vs tumours. Note: wildtype larvae can only be starved for 24 hours, given that they reach wandering stages 24 hr post CW. Critical Weight also is slightly different for wildtype and tumour bearing animals (WT: 60hALH; TUM: 68hALH), thus we have been subjecting WT and tumour animals to NR at 60h and 68h ALH respectively (Fig 2A-D, G; and Fig 6A-J).
b. When we compare the effect of NR or dietary manipulation between tumour animals of different genotypes, we subject them to NR or dietary manipulations for 48 hours post CW – Fig 2E-F, H, and Fig3A-G.

To make this clear, we include an explanation of our NR strategies in Line 133-135. To ease the reading of the paper, we have now simplified the schematics and added the precise timing of the starvation regime to the figure legends.

We agree that the -Y or -GP experiment (Fig 3A-G) should be done from 72-120hALH, we have redone the experiment and replaced the previous dataset (48-120hALH) with this new dataset (Fig. 3A-G).

**Figure 3 A-G – updated MS**

This different NR strategy led to slightly different results, mainly due the fact we are subjecting the animals to a shorter period of dietary manipulation at a point later in development, thus we saw weaker effects, however, our key message is that only yeast dropout is important to glial number and tumour growth. Thus, the different experimental regime supports our conclusion.

In addition, while you don't need to do everything, you should try something suggested here:

5- To strengthen the mechanistic model,

5.1. you should perform Path overexpression

We performed Path overexpression and examined BCAA levels, this data is now in Fig 4N and Fig S9, see ESSENTIAL point 1.

5.2. Do BCAA supplementation experiments in the context of PI3K or Ilp6 inhibition to determine whether BCAAs can rescue the glial and tumor phenotypes.

1) to decipher the relationship between PI3K, BCAA and Path, we performed further epistasis tests. We found that Path overexpression partially rescued the defects in glial expansion and tumour growth, caused by InR inhibition in the BBB glia (new data is incorporated into Fig 5N-R). This suggests that Path lies downstream of the PI3K to regulate glial and tumour growth.
2) To test if PI3K inhibition in the BBB alters brain BCAA, we performed LC-MS (Reviewer Figure). However, upon BBB InR inhibition, we did not see a significant reduction in brain BCAA levels, thus, we did not perform BCAA supplementation under InR inhibition as suggested by the reviewer. InR targets many other metabolic pathways, as well as regulate general cell anabolism (including translation), it is possible that InR does affect BCAA transport, but we could not detect it in our LC-MS due to these confounding factors.

**Reviewer Figure 1**: Quantifications of the normalised (to *lacZ^OE^*) AA concentration in *elav^C155^QF>QUAS-prosRi* tumour brains, where *lacZ^OE^* or *InR^DN^* was overexpressed in BBB glia using *GMR85G01-G4* (n = 10, 10).

5.3. Conversely, it would be important to test whether the effects of these pathways are diminished or abolished by path knockdown, thereby establishing Path as a functional mediator.

To test if the readouts of Insulin signalling is altered by Path knockdown, we examined foxo-GFP levels. We found that Path knockdown caused decreased nuclear foxo-GFP (S10B-D Fig), indicating that the PI3K pathway is in fact activated upon path knockdown, and that Path is likely to be downstream of the PI3K pathway.

To strengthen our model that the mTOR-S6K pathway lies downstream of Path, we repeated the epistatic experiments in Fig 7K-N with two appropriate controls (*lacZRi; mCherryRi* and *lacZRi; S6K^CA^*) and with the BBB glial driver (*GMR85G01-G4*). This new data is included as Fig 7K-L to replace the previous data (as shown below).

**Fig 7K-L – updated MS**

In our previous MS Fig 7H-J, we showed that RagCA or S6KCA activation using pan-glial driver repo-G4, rescued both glial cell number and tumour growth. To strengthen our model, we repeated this experiment using the *GMR85G01-G4* driver (Fig7H-J, updated MS). This new data supports our model that Path-mTOR-S6K pathway in the BBB glia is important in sustaining brain tumour growth.

**Fig 7H-J in updated MS**

5.4. Additionally, measuring BCAA levels under these genetic conditions would help directly link pathway activity, Path expression, and amino acid availability. Without such functional validation, the proposed model connecting upstream signals to tumor growth via Path-BCAA regulation remains speculative.

Please refer to ESSENTIAL point 1 and 5.1.

**Reviewers' comments**

Rev. 1:
Dong et al. present a study identifying the amino acid transporter Pathetic (Path) at the blood-brain barrier (BBB) as a key regulator of brain tumor sensitivity to nutrient restriction (NR), revealing the molecular role of the perineurial glial BBB cells in controlling tumor growth. The authors provide strong evidence that neuroblast tumors lose the ability to buffer against NR through the path transporter. However, the manuscript lacks sufficient experimental evidence to fully support its mechanistic claims that the NR buffering capacity is due to brain BCAA levels. Several points are listed below that would strengthen the conclusions if addressed:

The evidence suggesting that Path levels correlate with brain BCAA levels could be strengthened. In Figure 4, the authors show that glial-specific knockdown of path under nutrient restriction reduces tumor growth and brain amino acid levels—particularly BCAAs—without affecting known Path substrates, suggesting a broader role for Path in regulating amino acid availability.

6. While path knockdown (KD) was assessed for its effect on brain amino acid concentrations, **no equivalent analysis was performed in the context of Path overexpression**, even though the authors show (Fig. 4I-J) that Path overexpression in PG cells partially rescues tumor growth under NR conditions. It remains unclear whether the observed phenotypic rescue correlates with restored levels of BCAAs.

This point has been selected as ESSENTIAL POINT to be addressed by the editor. Please refer to our answer to point 1.

From Figure 5-7, the authors explore the upstream regulation of Path through the PI3K/AKT and Ilp6 signaling pathways, suggesting a mechanistic framework for how nutrient restriction downregulates Path expression. However, the link between these signaling mechanisms and the functional regulation of BCAA levels or transport remains insufficiently tested.

7. While Figure 7 shows that path knockdown impairs tumor growth and that its expression is necessary for tumor recovery, it remains unclear whether the upstream pathways act specifically through Path and BCAA availability. **To strengthen the mechanistic model, I suggest performing Path overexpression and/or BCAA supplementation experiments in the context of PI3K or Ilp6 inhibition to determine whether BCAAs can rescue the glial and tumor phenotypes.**

**Conversely, it would be important to test whether the effects of these pathways are diminished or abolished by path knockdown, thereby establishing Path as a functional mediator.**

**Additionally, measuring BCAA levels under these genetic conditions would help directly link pathway activity, Path expression, and amino acid availability. Without such functional validation, the proposed model connecting upstream signals to tumour growth via Path-BCAA regulation remains speculative.**

These three subpoints under point 7 have been selected as essential points to address by the editor in point 5. Please refer to our answer to point 5.

8. The authors assert that Path is a BCAA transporter Lines 460-461 "these data suggest that PG Path, rather than transporting its known substrates, likely regulates glial and tumour growth by controlling the transport of other AAs, including BCAAs.", **but the evidence for this is weak. If the authors are shifting the known paradigm, they should provide evidence for BCAA transport through Path.**

We agree that we don’t have evidence showing BCAAs are directly transported through Path. From the data we have, we can only conclude that Path regulates the availability of BCAAs and many other AAs, including Methionine, Threonine and Serine (Fig 4M).

To examine the transport properties of Path, Goberdhan et al. has overexpressed Path in *Xenopus oocytes,* a system with no basal Path expression. They showed that Path has high affinity for Alanine (Goberdhan et al., 2005). This contradicts with discoveries made in our system, where we observed no reduction of Alanine upon Path knockdown in the BBB. So, the role of Path in regulating transport or availability of AAs might be context specific. It has also been shown that Path knockdown in WT brains, FB, or SGs does not cause any obvious growth defects (Lin et al., 2015). Again, this suggests Path regulates AA availability in a context-specific manner.

Lastly, as the *in vitro* system showed results that contradicts our *in vivo* findings, and due to technical limitations in assessing Path transport properties *in vivo*, we are unable to experimentally test this point. We have revised the text to tone down our conclusions.

9. The authors use the Fly-FUCCI system driven by the PG-specific GMR85G01-GAL4 driver to monitor cell cycle dynamics in perineural glia (PG) under fed and NR conditions. They report a significant increase in G1-phase cells and a corresponding decrease in S-phase cells under NR, suggesting a slowdown or arrest of the cell cycle. The authors also state that this change is not due to increased cell death, thereby implying true cell cycle arrest. However, the FUCCI system alone is insufficient to conclusively demonstrate cell cycle arrest. FUCCI provides a proxy for cell cycle phase distribution but does not differentiate between transient delay and true checkpoint-mediated arrest. To substantiate the claim of cell cycle arrest, I suggest performing additional experiments such as analysing the expression of phase-specific markers (e.g., Cyclins) and examining well-established cell cycle arrest or checkpoint markers, including p21, p27, phosphorylated Chk1/Chk2, or γH2AX in the
case of DNA damage-induced arrest**.**

This point has been selected as ESSENTIAL POINT to address by the editor in point 2. Please refer to our answer to point 2.

10. For statistical analysis, it seems that fed was compared to NF; however, within-group analysis (fed control compared to KD or OE) is not shown for some of the plots. This can result in misinterpretation of the results (e.g. Fig 2S-T). Based on the data, it seems that CDk4^OE^; CycD^OE^ have increased glial numbers independent of fed/NR.

This point has been selected as essential point to address by the editor in point 3. Please refer to our answer to point 3.

11. It is not entirely clear why the NR timelines are different between figures and even between WT and tumor (e.g. Fig 2 & 4 NR was from 72-96h while in Fig 6. The WT was NR from 65h to 92h while tumor was from 68h to 92h). Fig 3, the =Y or -GP was fed for much longer (48-120h). It is difficult to make comparisons among the groups this way.

This point has been selected as essential point to address by the editor in point 4. Please refer to our answer to point 4.


Minor comments:
12. In Figure 4M, the authors report that BCAA levels are reduced following path knockdown, supporting the idea that Path modulates amino acid availability. While this trend is generally supported by the data, it should be noted that isoleucine (Ile) does not show a statistically significant decrease, which somewhat weakens the claim of a consistent BCAA reduction.

It is true with the dataset shown in Fig 4M that there is a weak reduction of Ile (but not significant). However, we have a repeat dataset (below), where we also observed the downregulation of Ile upon Path knockdown in the BBB, thus supporting our claim that BCAA levels are reduced upon Path knockdown.

13. Notably, lysine (Lys) levels are nearly doubled following Path knockdown, yet this unexpected increase is not addressed or discussed in the manuscript. Can the authors comment on this finding?

Indeed, we observed an increase of Lys levels following Path knockdown, furthermore, Lys level also increased upon the expression of InR^DN^. We think it is possible that path or InR^DN^ might upregulate the expression of other transporters such CATs (Zaragozá 2020), that can mediate Lys transport. It is also possible that Lysine catabolism (by LKRSDH) is downregulated, which leads to increase of Lysine levels (St Clair et al. 2017). We have added this discussion to our manuscript.

14. Please define nutrient restriction. Is it complete starvation?

We now defined this term at Line 111-112.

14. Is Path expression in the brain restricted to PG cells? How about its expression in the SPG?

Path is expressed in all the glia subsets (Feng et al.,2020). It is also expressed in the SPG (Reviewer Figure 2). And we found that Path knockdown using a SPG driver (moody-G4) also reduced tumour growth (Reviewer Figure 3).

**Reviewer Figure 2. PathGFP is expressed in SPG.**

(A): Surface-section image of late L3 wildtype larval brain lobe, where *moody-G4>UAS-mRFP* was used to mark SPG membrane and assess the expression of Path-GFP in SPG. Scale bar = 50μm. (B-C’’) zoomed-in images of (A). Scale bar = 20μm. (D): Deep-section image of late L3 wildtype larval brain lobe with *moody-G4>UAS-mRFP, Path-GFP*. Scale bar = 20μm. (E-E’’): zoomed-images of (D). Scale bar = 10μm. In (C-E), yellow arrows point to SPG membrane where Path-GFP is highly expressed and grey arrows point to SPG membrane where Path-GFP is lowly expressed.

**Reviewer Figure 3. Path is required in SPG to regulate brain tumour growth.**

Temporal knockdown of Path from 72 – 120 hr ALH in the SPG using *moody-G4* caused a reduction in tumour volume (as measured by Dpn voxels, green) in *elav-QF>QprosRi* brain lobes. Scale bar = 50μm

15. The title "…SCL36 amino acid transporter Pathetic…" should be "…SLC36 amino acid transporter Pathetic…"
We have corrected the mistake.


Rev. 2:

Background
Normal development of an animal depends on the availability of nutrients, including amino acids. Upon moderate nutrient deprivation undersized adults form with all organs formed isometrically with body size. However, when nutrient restriction occurs late in development, a preferential growth of the CNS is observed at the expense of other organs. This process has been termed brain sparing and occurs in humans as well as in flies. The Drosophila amino acid transporter pathetic (path) was previously found to be needed for overall body growth and dendrite growth (PMID: 26063572). The Path transporter is a member of the SlC36A family which transports proline, alanine, or tryptophan. A few years ago, path was found to be required for neural stem cell proliferation under nutrient restriction (PMID: 32741363). While Path is expressed in several different glial cell types as well as in neuroblasts, the glial expression domain is responsible for stem cell proliferation under nutrient
restriction (PMID: 32938923). The nutrient status also affects tumor growth in a Path dependent manner. When the oncogenic form of Ras, RasG12V is expressed in the eye disc of C-terminal Scr kinase mutant larvae, tumors develop. Strikingly, when such animals are raised on a high-sugar diet tumor formation is enhanced and muscle wasting is observed. The excessive tumor growth and the muscle wasting depends on circulating proline and the Path transporter.

During normal larval development previous work of L. Cheng (PMID: 21816278) defined the critical weight period after which brain sparing is observed under nutrient restriction. In the present paper the group now extends these old findings and asks how growth of brain tumor is affected by nutrient restriction.

16. In wild type larvae, nutrient restriction does not affect brain growth (Fig1D-F, here shown by anti-Mira/pH3 staining). When neuroblast division is triggered by QF/QUAS dependent silencing of prospero, brain tumors develop that are sensitive to nutrient restriction (Fig1G-I, here show by anti-Dpn staining). Importantly, brains stay small.

**Whether or not brain sparing is affected is not easy extractable from the Figures**, since different antibodies are used, and the scale bars are hard to read.

To address reviewer’s concern, we repeated the experiment and performed NR on WT and tumour bearing larvae post CW for 24 hours (72-96hALH), staining for Dpn, Repo and pH3. We showed that both glial number and NB total volume are significantly reduced under NR in the tumour context (Fig 1D-I, and Fig 2A-D and G). We also showed that the percentage of NBs undergoing mitosis is reduced under NR in the tumour and not in the control (Fig 1I). Together, **these data indicate that, unlike wildtype brains, brain tumours are not spared under post CW NR**. We now moved the Fig 1D-F to S1A-D and removed S2A-C Fig (repetitive data). And the new data is incorporated to Fig 1D-I.

Fig1 D-I in updated MS

17. The authors then show that reduction in tumor growth is due to a slowdown in cell cycle progression and that nutrient restriction can also affect glial tumor growth, but not epithelial tumors induced by scribble knockdown ( note there is a typo **the gene is called scribble not scribbled**) the eye imaginal disc.

We have corrected the mistake.

18. In a next step the authors analyzed whether nutrient restriction affects the number of glial cells and stained larvae for glial nuclei using the Repo marker.

**In both the tumor model as well as in wild type brain, nutrient restriction results in a reduction of the overall number of glial cells (What are the actual numbers?)** Please see the plot for the actual number below. Normalised data is displayed in Fig 2G for consistency.

**19.** **While this is clearly documented, no attempts are made to determine the glia cell type affected.** To address this question, we also characterised the effect of NR on SPG nuclei number and size (S3G-J Fig) as well as CG number (S3K-M Fig).

**20**. The authors only use Fly-FUCCI and a driver that is specific to adult perineurial glia (**the expression pattern in the larva is not shown, is there no neuronal expression?)**

We thank the reviewer for the suggestion. To address reviewer’s question, we examined the expression pattern of *GMR85G01-G4* in the larvae, including in the gut, fatbody, salivary gland, wing discs and brains (S4E-H Fig). We observed that *GMR85G01-G4>UAS-* *stinger* does not colocalise with the neuronal marker Elav (S4A-A’’’ and D Fig). However, this driver is also expressed in the SPG, so, we further characterised the role of Path in the SPG, where we found that Path knockdown in the SPG using *Moody-GAL4* also caused a reduction in tumour growth (See point 14). Given that *GMR85G01-G4* is expressed in both PG and SPG during larval stages, we now describe this driver as the BBB glial driver and changed our conclusion accordingly.

21. supports the notion that these glial cells are affected (21-1) **but they do not show a specific role of perineurial glia**. (21-2) **One must count the numbers of the different glial subtypes in the different settings using the tools that are available** .

For 21-1, We showed in Fig 2Q-V that by sustaining BBB glial number under NR, we are able to spare tumour growth. This data already indicates the role of BBB glia in regulating tumour growth. For 21-2, we assessed the number of CG and SPG under fed vs NR, as explained in point 19.

22. Next the authors analysed amino acid transporter expression in fed and nutrient-restricted tumor bearing larvae. Under nutrient restriction, three amino acid transporters Minidiscs (MND), Juvenile hormone Inducible-21 (JhI-21) and Pathetic (Path), are transcriptionally downregulated. **Where these transporters are expressed is not mentioned**?

We thank the reviewer for the suggestion we have added this information to the manuscript.

23. **I failed to see arguments, however, supporting the conclusion that branched-chain amino acids "play a key role in regulating perineurial proliferation and, consequently, tumor growth".** We showed in Fig 3I-N that dropping out Leu, Ile and Met affected tumour size and glial number. To understand how BCAAs affect NB tumour growth, we included new data, showing Leucine tRNA synthase is downregulated in the tumour under NR, and is necessary for tumour expansion, Fig 3P-S and Fig S6).

24. Instead of following up on the role of MND and Jhl-21 the authors turn to an analysis of Path. Path is expressed by glia as well as in neuroblasts **(the latter not being mentioned in the paper).** Thanks for reviewer’s suggestion, we now describe its expression in NBs in Line 439-440).

25. **Unfortunately, all stainings of Path::GFP are of very low quality that do not allow any conclusions on the effect of nutrient restriction on path expression.**

To address this concern, we have now provided zoom-in images in Fig 4A-B, Fig S7A-B, D-E and G-H, S8A-B Fig, Fig 5E-G. In addition, we added more explanation on how we quantify PathGFP intensity, Line 948-951).

26. In addition, to immune fluorescent imaging data, western blots must be used to demonstrate these claimed expression changes.

Unfortunately, WB is not going to help resolve this question, due to the fact that BBB glia represents only a small population of all cells in the brain.

27. **Why do the images in S5 FigA'/B' look so different to what is shown in S5 FigJ,K** ?

In S5A-B Fig, the images shown are middle section plane (Z6). To make this clear, we include a schematic showing the displayed brain section (S7A-C Fig, updated MS).

In S5J and K Fig (previous MS version), we showed the brain lobe surface plane (Z0) instead of the middle plane. This is because we don’t have ways to mark the glial membrane in the experiment in S5J and K Fig, so, we only claimed that “Path levels were also drastically reduced at the brain surface of NR-sensitive *brat RNAi* tumours after NR”. We have now removed this data.

Updated S7 Fig

28. To further deduce the role of path in perineurial glia the authors performed repo-Gal4 based knockdown experiments. The knockdown efficiency is documented in S6 FigA. **Here is a mismatch between the image shown and the quantification shown in S6 FigC.**

**The quantification in S6C Fig did support what is displayed in the representative images in S6A-B Fig (yellow arrows, as shown below).**

Moreover, **the perineurial glia Gal4 driver *GMR85G01-Gal4* must be used in this control experiment to judge whether there it can induce any change in path expression.**

**To address Reviewer’s concern, we have now used *GMR85G01-G4>mRFP* in this experiment (now S8A-C Fig).**

**29. The statement that nutrient restriction induces a differential regulation of path expression in glia depending on the presence of brain tumors is fascinating - but unfortunately the overall low quality of the images makes it hard to believe. Add western blot experiments.**
We provided zoomed-in images in Fig 4A-B, S7A-B, D-E and G-H Fig, S8A-B Fig, Fig 5E-G, and include BBB markers for accurate quantification. Unfortunately, western blotting will not tell us how cell-type-specific changes occur under NR, as BBB cells only account for a small percentage of all brain cells.

30. Gain of function of path is claimed to override the effect of nutrient restriction in glial and tumor growth. **What is the control here?** Control: *elavQprosRi; GMR85G01-G4 x w1118*. We have added this information to the figure legend.

31. Is the effect of Path overexpression the same as observed to what is noted upon Path knockdown? The effect of Path.A overexpression on glial number and tumour size is not the same as path knockdown. Path overexpression increased the sparing under NR.

32. Quantification of brain amino acid concentrations indicates a decrease in the amount of branched-chain amino acids but not the ones that are normally transported by Path - but which are transported by MND and JhI-21. The interpretation is that Path regulates transport of such amino acids. This is an interesting possible conclusion but instead of addressing it in more details the authors switch to the analysis how Path expression is regulated and analyzed PI3K signaling that eventually leads to a regulation of the nuclear localization of FOXO. **Again, I must confess that I was not convinced by the data shown that in nutrient restricted larvae FOXO localizes more to nuclei.** To address this concern, I replaced the low-mag representative images in Fig. 5B-C with 4 zoomed-in images, clearly showing nuclear localisation of FOXO-GFP under NR vs Fed.

Fig 5B-D in updated MS

**33. Likewise, the analysis as well as the description of the experiment shown in Fig 5E-H is not convincing. The reduction of Path could be judged from the images BUT why is the repo-RFP signal then different in every image? And how can a pan-glial expressed RFP marker serve as surface glia marker?** The claim that nutrient restriction downregulates PI3K signaling in perineurial glia is not supported by the data.

To address this concern, we repeated this experiment using *GMR85G01-G4>UAS-mRFP* to mark the BBB and modulate the PI3K pathway (Fig 5E-H). However, as *GMR85G01-G4* is also expressed in the gut, activation the InR using this driver causes some systemic effect. We observed that the larvae were smaller than the controls. Therefore, we also kept and moved the data with *repo-G4* (originally in Figure 5E-H) to S10A Fig.

**Fig 5E-H in updated MS**

**Reference:**

St Clair, S. L., H. Li, U. Ashraf, J. A. Karty, and J. M. Tennessen. 2017. "Metabolomic Analysis Reveals That the Drosophila melanogaster Gene lysine Influences Diverse Aspects of Metabolism." *Genetics* 207 (4): 1255-1261. <https://doi.org/10.1534/genetics.117.300201>. <https://www.ncbi.nlm.nih.gov/pubmed/28986444>.

Zaragozá, R. 2020. "Transport of Amino Acids Across the Blood-Brain Barrier." *Front Physiol* 11: 973. <https://doi.org/10.3389/fphys.2020.00973>.
